# Time Course of Functional Connectivity in Primate Dorsolateral Prefrontal and Posterior Parietal Cortex during Working Memory

**DOI:** 10.1371/journal.pone.0081601

**Published:** 2013-11-19

**Authors:** Fumi Katsuki, Christos Constantinidis

**Affiliations:** Department of Neurobiology and Anatomy, Wake Forest School of Medicine, Winston-Salem, North Carolina, United States of America; University of Michigan, United States of America

## Abstract

The dorsolateral prefrontal and posterior parietal cortex play critical roles in mediating attention, working memory, and executive function. Despite proposed dynamic modulation of connectivity strength within each area according to task demands, scant empirical data exist about the time course of the strength of effective connectivity, particularly in tasks requiring information to be sustained in working memory. We investigated this question by performing time-resolved cross-correlation analysis for pairs of neurons recorded simultaneously at distances of 0.2–1.5 mm apart of each other while monkeys were engaged in working memory tasks. The strength of effective connectivity determined in this manner was higher throughout the trial in the posterior parietal cortex than the dorsolateral prefrontal cortex. Significantly higher levels of parietal effective connectivity were observed specifically during the delay period of the task. These differences could not be accounted for by differences in firing rate, or electrode distance in the samples recorded in the posterior parietal and prefrontal cortex. Differences were present when we restricted our analysis to only neurons with significant delay period activity and overlapping receptive fields. Our results indicate that dynamic changes in connectivity strength are present but area-specific intrinsic organization is the predominant factor that determines the strength of connections between neurons in each of the two areas.

## Introduction

The ability to allocate neural resources flexibly, to maintain and manipulate information in mind in accordance with the behavioral needs of that moment, is an essential aspect of intelligent behavior and an essential part of working memory models [1]. Neurophysiological studies using non-human primates readily reveal persistent neuronal discharges following the presentation of sensory stimuli that subjects were required to remember [2]. This persistent neuronal activity is tuned to specific properties of stimuli and is commonly considered a neural basis of working memory [3], [4]. Recurrent connections between layer II/III cortical neurons are thought to be critical in the generation of persistent discharges. Neurons that are activated by the appearance of a sensory stimulus continue to produce reciprocal excitation through a dense network of synaptic connections, allowing activity to be prolonged even after the disappearance of the original stimulus [5]. The dorsolateral prefrontal cortex (dlPFC) is reported to have an extensive network of intrinsic connections that could provide a neural substrate for persistent activity [6].

Persistent activity related to working memory was initially observed in the dlPFC, however, neural correlates of working memory have also been described in other brain areas including the posterior parietal cortex (PPC), a brain area that also plays a role in higher cognitive functions [7]–[12]. One of the major functional differences between the two areas is that neurons in the PPC typically represent the location of the most recent stimulus in the environment regardless of behavioral relevance [8], [13], whereas the dlPFC neurons are more likely to represent the behaviorally relevant stimulus even when distracting stimuli are presented [14]–[16]. The underlying basis of specialization of these two areas is an area of active research. Previous studies suggested a contribution of dopaminergic innervation in dynamically enhancing working memory in the face of distraction, which is widely recognized as one of the characteristics of dlPFC circuitry [17]–[20]. Dopamine inputs can enhance the conductance of NMDA receptors, ultimately improving the signal to noise ratio of information represented in persistent discharges [21]–[24]. Computational modeling studies have suggested that NMDA receptors are critical, as presence of both fast positive feedback and slow negative feedback in the system could lead to dynamic instability, disrupting persistent activity [25]–[27]. These models indicate that dynamic stability could be achieved by a slower excitation [26], [27]. The slow time constant of the NMDA receptor fits well this function, maintaining the postsynaptic neuron in a depolarized state for a prolonged period and thus promoting persistent activity [28]–[30].

Although it has been speculated that dopamine plays a role in dynamically strengthening recurrent connections between PFC neurons during working memory, very little experimental data are available on the time course of effective connectivity. A recent study revealed systematic differences in the geometry of intrinsic connections between PFC and PPC neurons; the strength of effective connectivity was stronger overall in the PPC particularly for neurons at distances ≤0.3 mm apart from each other [31]. This finding may be viewed as contrary to predictions about the role of dopamine in the prefrontal cortex. Since effective connectivity can be modulated dynamically [32], it is still possible that dopaminergic action can increase connectivity between neurons specifically in the dlPFC during the delay period of working memory, providing stronger resistance to distracting stimuli. Therefore, we hypothesized that effective connectivity would be higher in dlPFC than PPC, specifically during the delay period of working memory tasks. In the current study, we analyzed the intrinsic connectivity in the separate working memory task epochs and compared them between the dlPFC and the PPC.

## Methods

### Ethics Statement

All surgical and animal-use procedures in the present study were reviewed and approved by the Wake Forest University Institutional Animal Care and Use Committee, following guidelines by the U.S. Public Health Service Policy on Humane Care and Use of Laboratory Animals and the National Research Council’s Guide for the Care and Use of Laboratory Animals.

### Animals and Surgical Procedures

Data from three male, rhesus monkeys (*Macaca mulatta*) weighing 5–12 kg were used for this study. Monkeys in our colony are pair- or single-housed (depending on compatibility), in the same room with other conspecifics, in caging meeting the federal requirements of floor space depending on the animals’ size. Their health, body weight, water intake, and food consumption were monitored daily by veterinary staff. The animals were fed monkey chow administered by the Wake Forest Animal Resources Program, and supplemented with food treats such as fruits, nuts, and fresh produce. Behavioral training was accomplished via fluid regulation; animals received the same minimum amount of fluids (>20 ml/kg body weight/day, computed over a weekly period), regardless of whether they performed the task or not. All surgeries were performed using aseptic techniques in an approved surgical suite. Anesthesia was first induced with ketamine (10 mg/kg) and maintained with isoflurane (1–3%) throughout surgery. Approved analgesics were delivered post-operatively for 3–4 days (Butorphanol tartrate, 0.05 mg/kg after surgery; Buprenorphine HCl, 0.02 mg/kg for days 1–3; Ketoprofen, 5 mg/kg beyond day 3, as needed), under the guidance of veterinary staff. After surgery, animals were allowed to recover for at least 1–2 weeks before starting of experiments. Animals were provided with environmental enrichment (e.g. foraging devices, toys, mirrors, novel foods, novel scents) overseen by the institutional environmental enrichment coordinator. None of the animals used in this study was sacrificed.

### Neurophysiology

**Figure 1:**
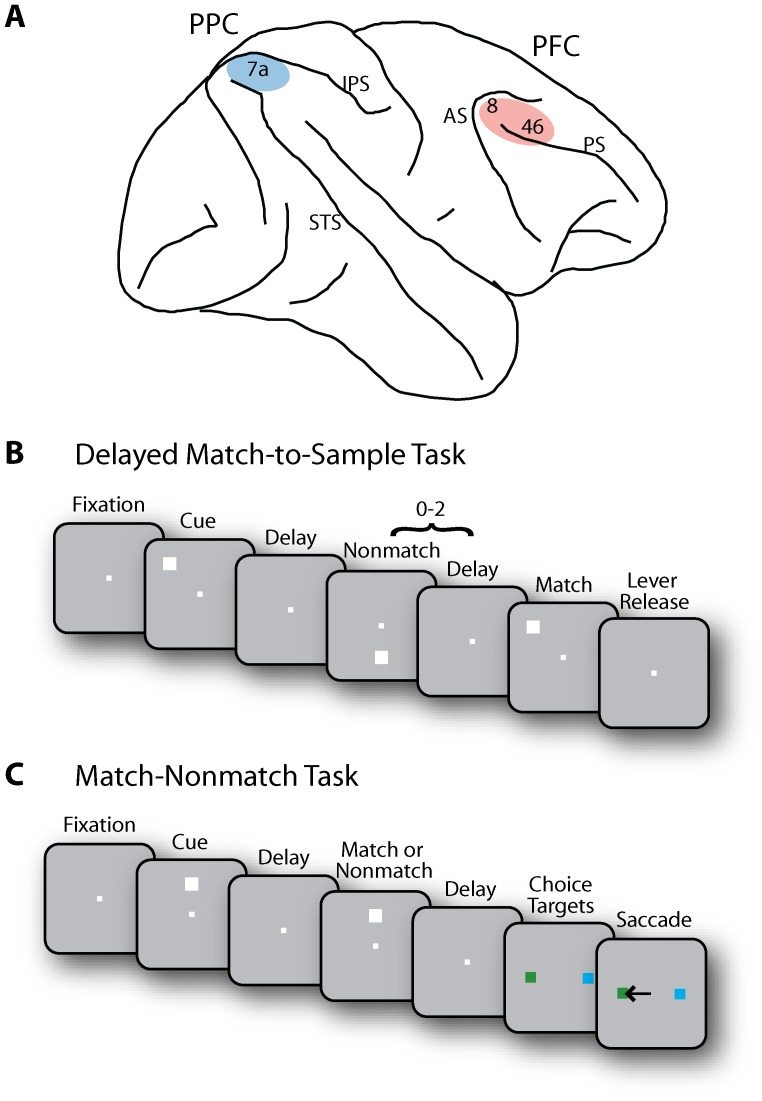
Brain areas and tasks. **A**) Schematic diagram of the monkey brain. The areas of recordings are highlighted. Abbreviations: AS, Arcuate Sulcus; IPS, Intraparietal Sulcus; PS, Principal Sulcus; STS, Superior Temporal Sulcus. **B**) Delayed Match-to-Sample task. Following the cue presentation, a match or non-match stimulus appeared. The monkeys were required to release a lever when a subsequent stimulus appeared at the remembered cue location. **C**) Match/Nonmatch task. Two choice targets were presented at the end of a trial. The monkey was required to saccade to a green target when the two stimuli were matching and to a blue target otherwise.

Surgical and neurophysiological procedures were performed as described in detail before [31]. Briefly, two 20-mm diameter recording cylinders were implanted over the dlPFC and PPC in each monkey (Fig. 1A). Neuronal recordings were performed using arrays of 2–8 microelectrodes in each cylinder, lowered into the cortex with a microdrive system (EPS drive, Alpha-Omega Engineering, Nazareth, Israel). The electrical signal was amplified, band-pass filtered between 500 Hz and 8 kHz, and recorded through a modular data acquisition system at 25 µs resolution (APM system, FHC, Bowdoin, ME). Neuronal recordings were performed in areas 46 and 8a in the dlPFC and area 7a in the PPC. Recordings from sulci were excluded to ensure that all data analyzed here were collected from the exposed surface of the cortex. Eligible neurons in the PPC were collected from the crown of the gyrus posterior to the intraparietal sulcus; neurons in the dlPFC were recorded from at least 1 mm away from the principal sulcus, in the superior convexity of the PFC, and in the surface area between the principal and arcuate sulci (Fig. 1A). To ensure that the analysis focused on horizontal connections across the surface of the cortex, three more selection criteria were applied: a) both neurons of a pair must have been recorded at a depth of <2.5 mm from the surface of the cortex; b) the two electrode penetrations of a pair must have met the surface of the cortex no more than 1 mm apart from each other (i.e. if one electrode went through more than 1 mm than the second before entering the cortex, the pair was eliminated); c) pairs of neurons that were recorded at depths >1 mm relative to each other were eliminated, even if the pairs were not located in the sulci.

### Behavioral tasks

The monkeys were positioned 60 or 68 cm away from a monitor in a dark room. An infrared eye position tracking system (model RK-716; ISCAN, Burlington, MA) sampled and recorded eye position at 240 Hz. The visual stimulus presentations were controlled by in-house software [33], developed in the MATLAB computational environment (Mathworks, Natick, MA). Two monkeys performed a Delayed Match-to-Sample task [34] and one monkey was trained with a Match-Nonmatch task [15], [35], shown in Fig. 1B and C. In the both tasks, monkeys were trained to remember the location of a cue and to indicate the remembered cue location by releasing a lever or by shifting gaze. A trial in the Delayed Match-to-Sample task consisted of a 0.5 s fixation period, a 0.5 s cue presentation, a 1.0 s delay, a pseudorandom sequence of 0–2 non-match stimulus presentations each lasting 0.5 s and separated by delay periods of 0.5 s, and a 0.5 s match stimulus presentation (Fig. 1B). In a trial of the Match-Nonmatch task, there was a 1 s fixation period, a 0.5 s cue presentation, a 1.5 s delay period, and a 0.5 s of a second stimulus presentation at the location identical (match) or diametrically opposite (non-match) to the cue location; following another 1.5 s delay period, two targets were presented, and the monkey was required to make a saccade to a green target if the two stimuli matched or to a blue target, otherwise (Fig. 1C). Although data were recorded in our previous studies using variations of these tasks, in the present study we only analyzed the data recorded with the spatial working memory tasks that involved single stimulus presentations and at least one delay period after the cue presentation.

### Neuron selection

Recorded spike waveforms were sorted into separate units using an automated cluster analysis method implemented in MATLAB called the KlustaKwik algorithm [36] which was based on principal component analysis of the waveforms. The results of the automated clustering were evaluated manually by experimenters and suspect waveforms were rejected from further analysis. We identified units with significant increase in firing rate during the presentation of visual stimuli by comparing the firing rate in the 0.5 s interval of a stimulus presentation with the 1 or 0.5 s interval of fixation (paired t-test; p<0.05). Neurons with a significant elevation of activity in other task epochs including the delay periods were evaluated in a similar way. Only trials with correct behavioral responses were used in the current analysis.

### Cross-correlation analysis

**Figure 2:**
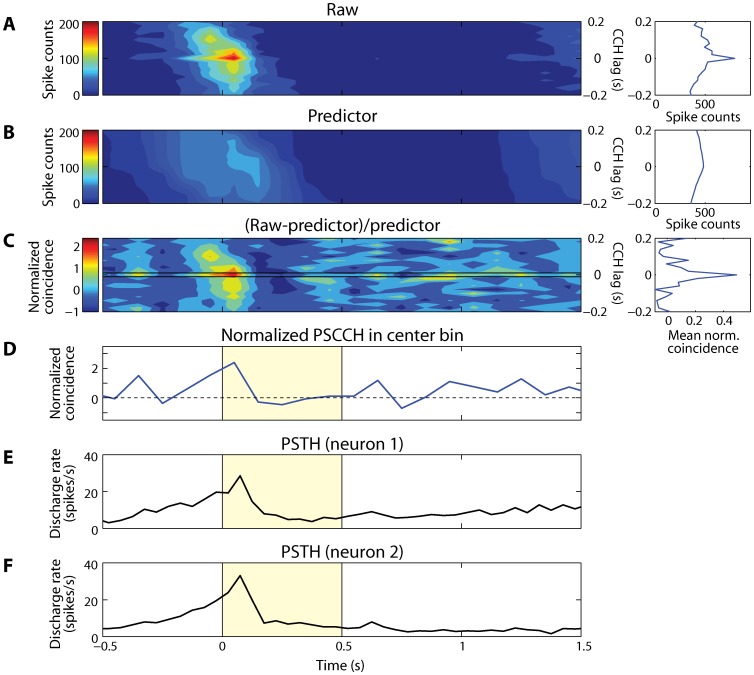
Peri-stimulus cross-correlation histograms (PSCCHs). **A**) Raw PSCCH of a dlPFC neuron pair. Horizontal bin size is 100 ms and vertical (CCH) bin size is 20 ms. The right panel illustrates a sum of the coincident spikes over time shown in the left panel. **B**) Predictor PSCCH of the same neuron pair. Predicted value of coincident spikes between the pair of neurons is plotted as a function of time. The predictor was estimated by correcting the raw PSCCH with a surrogate spike train method in which the spike time was randomly resampled (see methods). The right panel illustrates a sum of the coincident spikes over time shown in the left panel. **C**) Normalized PSCCH. The raw PSCCH was normalized by subtracting predictor and divided by predictor, bin-by-bin. Horizontal two lines represent the edges of center 20 ms bin. The right panel illustrates an average of normalized coincident spikes over time shown in the left panel. **D**) Normalized coincident spikes occurred within the center bin depicted in C. Yellow shaded area represents the cue presentation time. **E-F**) Raw peri-stimulus time histograms (PSTHs) of two dlPFC neurons separately. Firing rate of each neuron in the pairs used to plot PSCCH in this example is plotted as a function of time. The bin size is 50 ms.

The strength of intrinsic effective connectivity of each brain area was estimated by performing a cross-correlation analysis [37] on pairs of neurons recorded simultaneously from separate electrodes spaced 0.18–1.50 mm apart from each other. Each neuron pair used in this analysis was constructed from the neurons recorded from different electrodes. Only neuron pairs with more than 1000 spikes in total, at least 100 spikes in each neuron, and spikes available in every 250 ms time bins were used for this analysis. Time-resolved cross-correlation histograms, or peri-stimulus cross-correlation histograms (PSCCHs) were constructed from the spike trains of each pair of neurons [38], [39]. For this analysis, we used spikes in 0.5 s non-overlapping windows, spanning the length of the trial. In each 0.5 s window we determined the lags of spikes of the first spike train relative to all the spikes in the second spike train and incremented the corresponding 20 ms bins in the cross-correlation histogram. This is equivalent to creating a series of 20 ms bins centered on each successive spike of the first neuron and accumulating counts of spikes from the second neuron in the bins, as they were slid through the data. In order to minimize the potential effects of stimulus presentations or other factors covarying during the time course of a trial that could simultaneously increase firing rates in both neurons of a pair, we corrected CCHs with a surrogate spike train predictor [31]. To destroy temporal structures, surrogate spike trains were created by randomizing the time of each spike in a trial by sampling a replacement spike time from a uniform distribution spanning 250 ms around the time of the original spike (Fig. 2A). Predictor CCHs were obtained by repeating this procedure for 1000 times and averaging the values (Fig. 2B). The normalized PSCCH for each pair of neurons was computed by subtracting this predictor CCH from the raw CCH and then dividing by the predictor CCH on a bin-by-bin basis (Fig. 2C). To investigate changes in the effective connectivity between a pair of neurons over time, we determined the normalized coincidence occurring within the center 20 ms bin of the PSCCH (Fig. 2 D). Data were also analyzed using a 20 ms off-center bin of the PSCCH. The off-center bin located either to the left (–30 ms to –10 ms) or right (10 ms to 30 ms) of the center bin of CCHs was used. The side with the greater absolute value of the normalized PSCCH from each pair was chosen for the analysis.

### Receptive-field assessment

We refined the cross-correlation analysis by estimating the effective connectivity between two neurons based on stimuli appearing in the receptive field of both neurons, in the receptive field of one, or of neither. Receptive fields were mapped in the context of behavioral task by presenting single stimuli at different locations. For the analysis, we first found the stimulus locations that elicited the lowest and highest cue period activity. Stimulus locations that elicited activity greater than the average of the firing rates recorded for these locations were deemed to appear inside a neuron’s receptive field. Each pair of neurons was categorized into 2 groups based on the receptive field combination; the two neurons with overlapping receptive fields or the two neurons with non-overlapping receptive fields (NOV). The spatial locations of neuron pairs with overlapping receptive fields were further divided into 3 groups: 1) locations at the intersection of two receptive fields (INT); 2) locations at the non-overlapping part of the receptive fields (XOR); 3) and locations outside of both receptive fields (OUT) [40].

## Results

The intrinsic, effective connectivity between pairs of neurons recorded simultaneously within the dlPFC and within the PPC was analyzed in a time-resolved fashion (Fig. 1A). Data from three monkeys trained to perform spatial working memory tasks (Fig. 1B, C) were included in the analysis.

### Database

We analyzed neuron pairs from different microelectrodes, separated laterally by 0.18 to 1.50 mm. In order to focus on horizontal connections in each area, we established a number of conservative criteria for the selection of neurons used for analysis. We thus only analyzed neurons recorded from the crown of cortical gyri; electrode penetrations that advanced into the principal sulcus, arcuate sulcus (the Frontal Eye Field), or intraparietal sulcus (Lateral Intra-Parietal area) were excluded (see methods for selection criteria). In addition, we identified neuron pairs located at depths <1 mm of each other (mean and standard deviation of depth difference between electrodes: 0.28±0.34 mm in the dlPFC, 0.56±0.59 mm in the PPC). Although depth estimates are approximate, more than 90% of neurons in our sample were recorded at depths <1 mm from the surface of the cortex, corresponding to the supragranular layers. In order to secure a sufficient number of spikes in each time window to perform time-resolved cross-correlation analysis, only neuron pairs were selected with more than 1000 spikes in total; at least 100 spikes in each neuron; and spikes available in every 250 ms time bin. The total numbers of neuron pairs that passed all the criteria and were used in the present analysis were 561 pairs from areas 46 and 8a of the dlPFC (24, 366, and 171 pairs from the three monkeys, respectively) and 169 pairs from area 7a of the PPC (28, 46, and 95 pairs, respectively). There was no significant difference in behavioral performance between the sessions of the dlPFC and the PPC recordings (t-test, p>0.6); the average performance of three monkeys (excluding errors due to breaks in fixation) was 92% in the PPC recordings (82, 95, and 97%, for the three monkeys respectively) and 93% in the dlPFC recordings (68, 96, and 95%, respectively)

### Time course of effective connectivity during working memory

To investigate whether the intrinsic effective connectivity changes over the time course of working memory tasks, we computed time-resolved cross-correlation histograms, also referred to as peri-stimulus cross-correlation histograms or PSCCHs [38], [39]. First, the raw PSCCH (Fig 2A) was constructed by computing a cross-correlation histogram (CCHs) for each time window (a 100 ms bin is used in Fig. 2 for illustration purposes; a 0.5 s bin was used for all other analysis). Second, to estimate the expected number of spike coincidences at each time point, we used a surrogate spike train method to obtain a predictor PSCCH by randomizing the time of spikes within a 250 ms window in each trial (Fig. 2B). This window size ensured a sufficient number of spikes in each bin to conduct the time-resolved cross-correlation analysis. Third, to obtain a measure of effective connectivity independent of spike counts at each time point, we calculated the normalized PSCCH by subtracting the predictor PSCCH from the raw PSCCH and dividing by the predictor PSCCH, bin-by-bin (Fig. 2C). Previous studies showed that when a cross-correlation peak is present, it is most often centered at time zero [31], [41]. Therefore, to observe changes in strength of the effective connectivity between two simultaneously recorded neurons, we initially focused on normalized coincidence of the center 20 ms bin (±10 ms) of the PSCCH (Fig. 2D).

**Figure 3:**
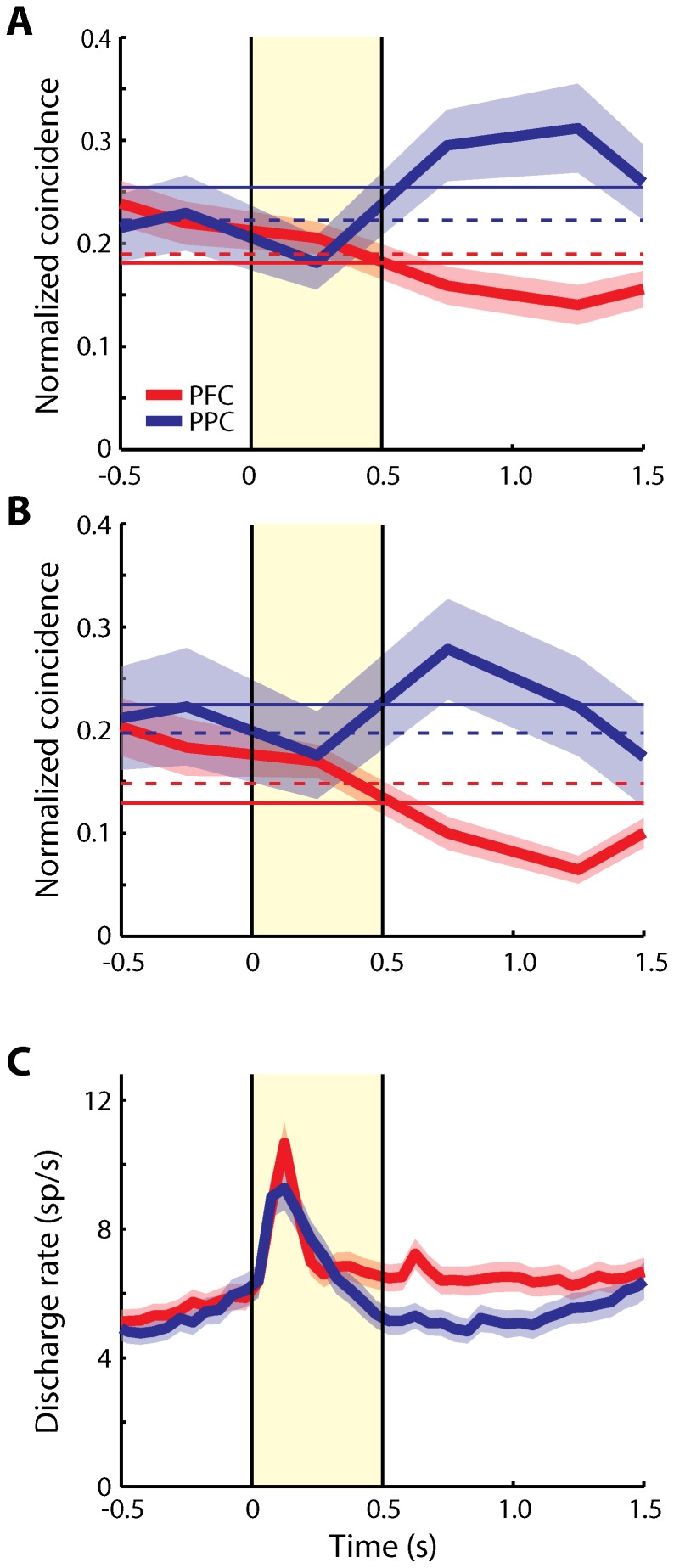
Time course of average effective connectivity. **A**) Average normalized coincident spikes occurring within the center bin of PSCCHs are plotted for the dlPFC (red) and the PPC (blue). Horizontal bin size is 500 ms. Samples include all neuron pairs regardless of significance in the delay period (dlPFC: 561 pairs, PPC: 169 pairs). Shaded area along each trace represents one standard error of mean computed across neuron pairs. Yellow shaded area represents the cue presentation period. Each horizontal solid line represents a mean over the period up to the first delay period depicted here (–0.5 s to 1.5 s). Each horizontal dotted line represents a mean over an extended trial period (–0.5 s to 4 s) including the match/non-match period. **B**) Average normalized coincident spikes occurred within the center bin of PSCCHs using the pairs with significant delay activity are plotted for each area (dlPFC: 265 pairs, PPC: 35 pairs). **C**) PSTH represents average firing rate of neurons used in the PSCCH analysis, including all neurons from the dlPFC (red, N = 403) and the PPC (blue, N = 178) with/without significant delay activity. The bin size is 50 ms. Shaded area along each trace represents one standard error of mean computed across neurons.

**Figure 4:**
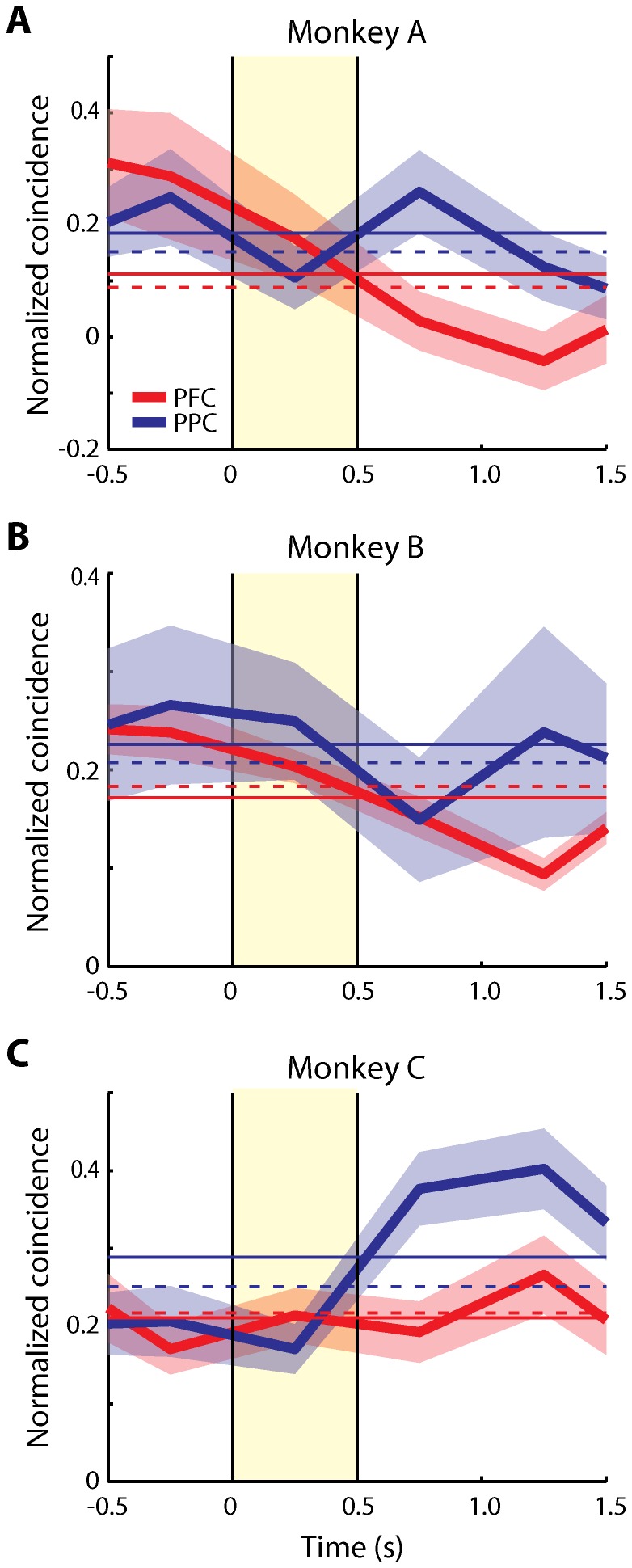
Time course of average effective connectivity for each monkey. Average normalized coincident spikes occurring within the center bin of PSCCHs are plotted for each monkey separately for the dlPFC (red) and the PPC (blue). **A**) Monkey A (dlPFC: 24 pairs, PPC: 28 pairs). **B**) Monkey B (dlPFC: 366 pairs, PPC: 46 pairs). **C**) Monkey C (dlPFC: 171 pairs, PPC: 95 pairs). Samples include all neuron pairs regardless of activity in the delay period. Horizontal bin size is 500 ms. Shaded area along each trace represents one standard error of mean, computed across neuron pairs. The cue presentation period is illustrated by the yellow shaded area. Horizontal solid lines and dotted lines represent a mean over the period up to the first delay period depicted here (–0.5 s to 1.5 s) and a mean over an extended trial period (–0.5 s to 4 s) including the match/non-match period, respectively.

**Figure 5:**
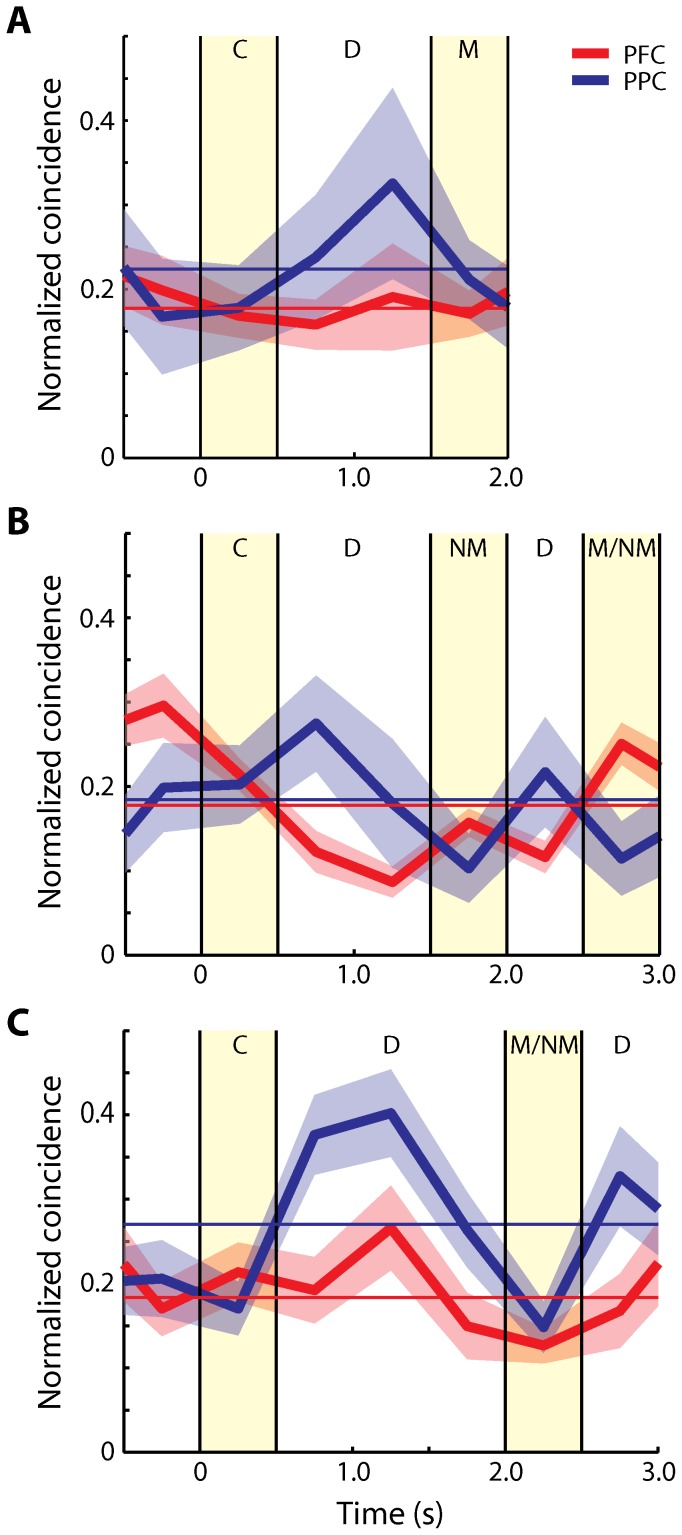
Time course of average effective connectivity for different trial types. Average normalized coincident spikes occurring within the center bin of the PSCCHs are plotted for the dlPFC (red) and the PPC (blue) for each trial type. Samples include all task-modulated neuron pairs. **A**) Trials in which cue period was followed by a match in the Delayed Match-to-Sample task (dlPFC: 225 pairs, PPC: 48 pairs). Shaded area along each trace represents one standard error of mean computed across neuron pairs. The first yellow shaded area represents the cue period, and the second one represents the match period. Horizontal solid lines represent the mean over the period depicted in the plot. **B**) Trials in which the cue period was followed by a non-match stimulus in the Delayed Match-to-Sample task (dlPFC: 371 pairs, PPC: 66 pairs). The first yellow shaded area represents the cue period, the second one represents the first non-match period, and the third one represents the second non-match/match period. **C**) Trials in the Match-Nonmatch task (dlPFC: 171 pairs, PPC: 95 pairs). The first yellow shaded area represents the cue period, the second one represents the second stimulus presentation period.

To obtain mean values of normalized, effective connectivity in each of the two areas across the time course of the trial, we repeated this analysis for all pairs of neurons with significant elevation of activity in any task period that met the minimum spike number criteria, and averaged across neurons. We first performed this analysis over the time interval that was common to all tasks and trial conditions, which included 0.5 s of fixation, the 0.5 s cue presentation and 1 s of delay period activity. Overall, normalized effective connectivity was higher in the PPC than the dlPFC in this sample of neurons, in agreement with our previous findings [31]. We did not observe any time interval where effective connectivity was significantly higher in dlPFC than PPC. The time course of effective connectivity was largely independent of firing rate changes; we observed little difference in effective connectivity between the fixation and cue presentation periods, when the maximum difference in firing rate was observed (Fig. 3). Interestingly, the effective connectivity was especially higher in the PPC than the dlPFC during the delay period (Fig. 3A, t-test, p<10^−5^). The effect was consistent across monkeys; higher effective connectivity for the PPC was observed during the delay period following the cue presentation in each of the three subjects (Fig. 4). Examining separately different task conditions (Fig. 5) confirmed that the effective connectivity of the PPC was consistently higher in the delay period following the initial cue presentation, as well as the delay period following a subsequent stimulus (Fig. 5B and C). This was contrary to expectations that effective connectivity may be enhanced in the dlPFC during the maintenance of working memory.

These results were obtained based on all available neurons. We next tested if this feature was maintained for the subset of neuron pairs that showed significant activity during the delay period, indicating that both neurons were active during working memory. There were 265 pairs from the dlPFC (2, 217, and 46 pairs from the three monkeys, respectively) and 35 pairs from the PPC (6, 12, and 17 pairs, respectively) for which both neurons had significant delay period activity. In this sample, too, we observed significantly higher effective connectivity in the PPC than the dlPFC during the delay period (Fig. 3B, t-test, p<10^−6^).

### Effect of firing rate

An important consideration in determining the effective connectivity is the effect of firing rate, since higher firing rate can increase the apparent correlation between two neurons [42]. We therefore wished to make sure that a difference in firing rate of neurons in the dlPFC and the PPC could not be responsible for the differences in strength of effective connectivity between the two areas that we observed. The mean firing rates of neurons analyzed in the current study were compared between the dlPFC and the PPC (Fig. 3C). The average firing rate of dlPFC was significantly higher than the PPC in the 1 s delay period (t-test, p<0.05), but no significant difference was found in the fixation and cue presentation periods. This suggests that the higher correlation strength observed in the PPC during the delay period was not due to the effect of firing rate.

### Effect of distance between neurons

Previous studies in different cortical areas demonstrated that strength of effective connectivity declined systematically as a function of horizontal distance between two neurons [43]–[45]. We reported in a prior study that the intrinsic effective connectivity of PPC neurons was higher than dlPFC neurons during the working memory tasks, particularly for the pairs with short (≤0.3 mm) horizontal distances between neurons [31]. Lower effective connectivity in the dlPFC during the delay period might have been observed due to unequally distributed distances in the two samples. We therefore evaluated if the distributions of neuron pairs across distances were similar in the dlPFC and the PPC. All pairs of neurons with significant elevation of activity in any task epoch that met a minimum spike number criterion were assessed. There were 42% of neuron pairs with distances ≤0.3 mm (234/561 pairs) in the dlPFC and 41% of neuron pairs in the PPC sample (69/169 pairs). Average normalized effective connectivity during the delay period was significantly lower in the dlPFC than the PPC for the ≤0.3 mm distance group (first 0.5 s of the delay period: 0.14±0.35 for dlPFC and 0.24±0.48 for PPC, t-test, p<0.05, second 0.5 s of the delay period: 0.13±0.39 and 0.28±0.70, t-test, p<0.05). Even for the pairs with distances >0.3 mm, for which overall difference in intrinsic effective connectivity was smaller between areas in the previous study, average effective connectivity during the delay period was still significantly lower in the dlPFC than the PPC (first 0.5 s of the delay period: 0.17± 0.49 for dlPFC and 0.33± 0.43 for PPC, t-test, p<0.01, second 0.5 s of the delay period: 0.15± 0.49 and 0.33± 0.44, t-test, p<0.01). When the mean effective connectivity was compared across neuron pairs grouped by the two areas using distance as a covariate, there was a significant difference in intrinsic effective connectivity between the two areas (ANCOVA, p<0.001). These results suggest that observed lower effective connectivity in the dlPFC compared to the PPC during the delay period was not due to the effect of distances between neuron pairs.

### Receptive field relationship

**Figure 6:**
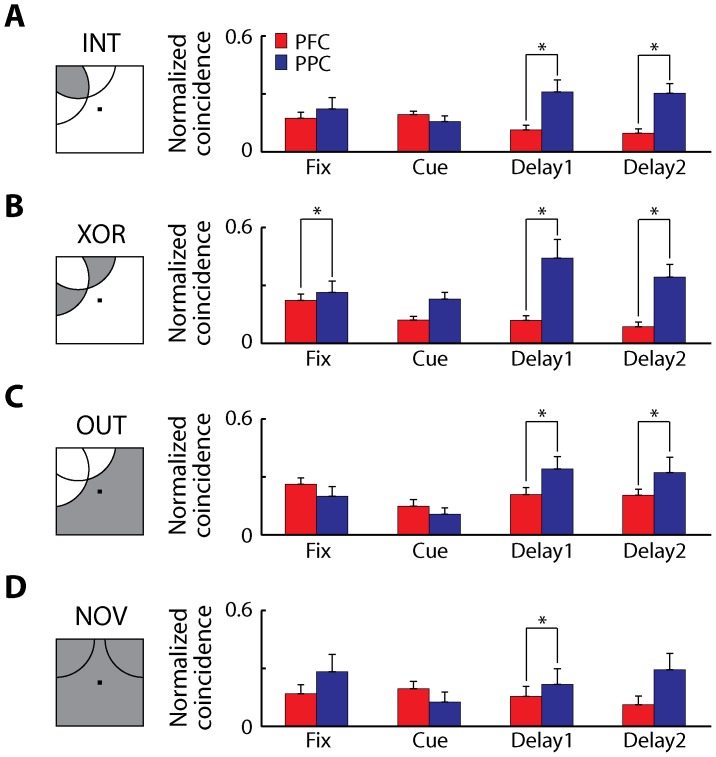
Effective connectivity and receptive field type. Average normalized coincident spikes occurring within the center bin of the PSCCHs are plotted for each receptive field type of pairs of neurons for the dlPFC (red) and the PPC (blue). Neuron pairs with/without significant delay activity were used in this figure. Each task epoch lasted 0.5 s; the delay period was divided into bins each lasting 0.5 s. Arcs in the insets represent the receptive field of each neuron, and gray shaded areas represent the stimulus location used for each analysis. **A**) Locations at the intersection of two receptive fields (INT, dlPFC: 313 pairs, PPC, 81 pairs). **B**) Locations at the non-overlapping part of the receptive fields (XOR, dlPFC: 291 pairs, PPC 101 pairs). **C**) Locations outside of both receptive fields (OUT, dlPFC: 306 pairs, PPC: 101 pairs). **D**) Pairs with no overlapping receptive-fields (NOV, dlPFC: 73 pairs, PPC: 34 pairs). Star marks represent significant differences between groups observed in post-hoc tests (Tukey's LSD, p<0.05 for all comparison).

It is still possible that stronger dynamic interactions are present in the dlPFC, but specific to neurons that closely share functional properties and therefore represent the same stimuli in memory. To compare more closely the difference in connectivity in two areas based on the separate task epochs and the stimulus preference of the neurons, we further grouped the data according to a type of receptive field combination of each pair of neurons [40]. Neuron pairs were first categorized into two groups based on their receptive field locations; pairs with overlapping receptive fields, or pairs with non-overlapping receptive fields (NOV). Stimulus conditions for the pairs with overlapping receptive fields were further separated into three categories: 1) stimulus locations at the intersection of two receptive fields (INT); 2) stimulus locations at the non-overlapping parts of two receptive fields (XOR); 3) stimulus locations outside of both receptive fields (OUT). We computed PSCCHs for each category and examined the connectivity of two areas for each task epoch (Fig. 6). We were particularly interested in the INT group (dlPFC: 313 pairs, PPC: 81 pairs), since this represents stimulus conditions that activated both neurons. For this group, too, the intrinsic effective connectivity was higher overall in the PPC than in the dlPFC (2-way ANOVA, p<0.001 for area, p>0.8 for epoch), and in fact significantly higher during the delay period of the task (t-test, p<0.001 for first 0.5 s of the delay, p<10^−4^ for second 0.5 s of the delay, Fig. 6A). Overall (Fig. 6A, B, C, and D), there were significant main effects of the area and the task epoch, but not of the receptive field type (3-way ANOVA, p<10^−6^, p<0.05, p>0.3, respectively). Post-hoc tests revealed that there were significant differences between the dlPFC and the PPC at the delay periods of the INT group (dlPFC: 313 pairs, PPC: 81 pairs), XOR group (dlPFC: 291 pairs, PPC: 101 pairs), OUT group (dlPFC: 306 pairs, PPC: 101 pairs), and the NOV group (dlPFC: 73 pairs, PPC: 34 pairs, Tukey's LSD, p<0.05 for all comparisons), but no significant difference was found in other task epochs, except the fixation period of the XOR group. These results indicated that even when examining separately trials that involved presentation of a stimulus in the receptive field of both neurons or not, the PPC exhibited higher overall intrinsic connectivity.

Finally, we considered the combined effect of significant delay period activity in both neurons of a pair and the location of stimuli receptive field. The intrinsic effective connectivity was again overall higher in the PPC than in the dlPFC for the INT group of neurons with persistent delay activity (24 and 188 pairs, respectively, 2-way ANOVA, p<0.01 for area, p>0.8 for epoch). Significantly higher effective connectivity in the PPC than the dlPFC was observed during the delay period (t-test, p<0.01 for first 0.5 s of the delay, p<10^−4^ for second 0.5 s of the delay). We found that there were main effects of the area, but not of the task epoch and the receptive field type (3-way ANOVA, p<0.01, p>0.5, p>0.6, respectively). Significant difference between the dlPFC and the PPC at delay periods were found in the INT group (dlPFC: 188 pairs, PPC: 24 pairs) and the XOR group (dlPFC: 152 pairs, PPC: 25 pairs, Tukey's LSD, p<0.05 for all comparison), but not in other task epochs and in the OUT group (dlPFC: 171 pairs, PPC: 29 pairs) and NOV group (dlPFC: 29 pairs, PPC: 4 pairs). These findings indicate a significant difference in dynamic effective connectivity in dlPFC versus PPC during working memory. Interestingly, the connectivity between neurons with the same stimulus preference was still lower in the dlPFC than the PPC during the delay period when the information of stimulus was held in a form of persistent activity.

### Off-center peaks

**Figure 7:**
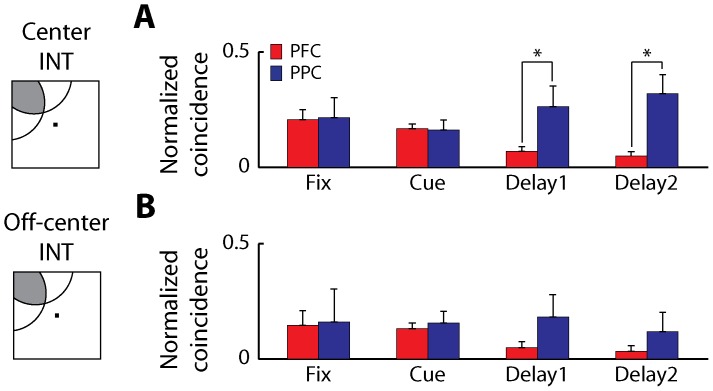
Effective connectivity of center bin vs. off-center bin. Average normalized coincident spikes occurring within the center 20-ms bin, and within a 20-ms off-center bin of the PSCCHs are plotted for the dlPFC (red) and the PPC (blue). The off-center bin used for each pair of neurons extended either between –30 ms to –10 ms or 10 ms to 30 ms of the CCH. Neuron pairs with significant delay activity are shown. Each task epoch lasted 0.5 s; the delay period was divided into bins each lasting 0.5 s. Analyses using locations at the intersection of two receptive fields (INT) are shown here. **A**) PSCCHs using the center bin (dlPFC: 188 pairs, PPC: 24 pairs). **B**) PSCCHs using the off-center bin (dlPFC: 171 pairs, PPC: 20 pairs). Stars represent significant differences between groups observed in post-hoc tests (2-way ANOVA and Tukey's LSD, p<0.05 for all comparison).

Peaks in individual CCHs we observed were most often centered at time zero, indicating common input as the dominant form of observable interactions between neurons we recorded from. However, there is still a possibility that systematic differences exist for off-center peaks between the two areas, underlying differences in connections where one neuron of the pair drives the second. A higher percentage of such peaks in the dlPFC than the PPC could mediate differential modulation of effective connectivity during working memory, due to the influence of dopamine or other factors. To investigate this possibility, we analyzed cross-correlation strengths based on off-center bins. We used a 20 ms bin located either at left side of the center bin (–30 ms to –10 ms lag) or right side of the center bin (10 ms to 30 ms lag) of CCHs; whichever of the two had a greater absolute value was chosen for each pair of neurons. The strengths of effective connectivity computed based on the center bin and the off-center bin were compared using the neurons with significant delay period activity (Fig. 7). When the stimulus was located at the shared receptive field of two neurons (INT), the effective connectivity computed with the center bin was significantly higher in the PPC than the dlPFC during the delay period (Fig. 7A, 2-way ANOVA, p<0.01 for area, p>0.8 for epoch, Tukey's LSD, p<0.05 for both delay1 and delay2). A similar trend was observed with the off-center bin analysis (Fig. 7B). The effective connectivity of PPC neurons was still higher than dlPFC neurons during the delay period although the difference between the areas was not statistically significant (Fig. 7B, 2-way ANOVA, p>0.2 for area, p>0.8 for epoch). The results indicate that no type of effective connectivity was stronger in dlPFC compared to PPC during the delay period of working memory tasks.

## Discussion

The present study demonstrates differences in the time-resolved intrinsic connectivity of the dlPFC and the PPC, two cortical areas critical for cognitive functions such as attention and working memory [46], [47]. We estimated connectivity between neurons based on the strength of the correlated firing at the millisecond scale. Therefore we refer to connectivity as “effective” [48], in contrast to anatomical connectivity. Our results demonstrated that effective connectivity varied during epochs of the task, independent of changes in firing rate. Our hypothesis was that effective connectivity would be higher in the dlPFC relative to the PPC during the delay period of working memory tasks. This was expected as a consequence of the action of dopamine and other factors that endow the PFC with unique properties, such as higher resistance to distractors. In agreement with a previous study which revealed differences in the geometry of intrinsic connections between the dlPFC and PPC [31], intrinsic connectivity differed between the two areas during various epochs of the working memory tasks. However, contrary to our hypothesis, significantly lower effective connectivity during the delay period was observed in the dlPFC compared to the PPC. This was true for neuron pairs with the same stimulus preference and when neuron pairs with significant delay period activity in both neurons were analyzed separately. The effect could not be attributed to systematic differences in firing rate or distances between neurons in the two samples. Although dopaminergic innervation in the dlPFC may facilitate persistent discharges during the delay period of working memory, the current measurements of functional connectivity did not demonstrate higher intrinsic connectivity in the dlPFC than the PPC for the memory maintenance period.

### Neuronal activity during working memory

It is well known that neurons in the dlPFC exhibit persistent activity during working memory tasks [3]. This persistent activity represents the properties of remembered stimulus such as spatial location, color, and shape [3], [35], [49]–[51]. PPC neurons are also reported to discharge in the delay period of working memory tasks [8], [52], [53], and similar to the dlPFC, responses of neurons in the PPC represent the spatial location of the remembered stimulus [11]. Other response properties such as percentages of neurons activated, response magnitudes, and temporal envelopes of responses are also comparable in the two areas [7], [15].

Response patterns in memory tasks requiring the maintenance of the original stimulus information with the presence of distracting stimuli have been revealing in regard to the properties of neural circuits involved in working memory. In this type of situation, PPC neurons generally represent the spatial location of the most recent stimulus, while PFC neurons can actively represent the original stimulus even after the appearance of behaviorally irrelevant distractors [8], [14], [15], [54]. When distractors appear in the receptive field of a neuron after presentation of an initial cue outside of the receptive field, the distractors elicit neuronal activity which may persist in the delay period, both in the dlPFC and PPC [8], [15]. However, distractor-generated activity appears to be filtered to a greater extent in the PFC, at least in the context of some tasks [16]. Similar to the PPC, the inferior temporal cortex has been shown to have a diminished representation of original stimulus following presentation of distractors during object memory tasks [54]–[57]. These results suggest the unique ability of the PFC to resist interference during working memory.

Although the aforementioned studies have emphasized activity that persists throughout the delay period after a stimulus, more recent work has revealed that information can also be represented in dynamic pattern of activity, so that stimulus properties may be present transiently during the delay period, both in the PFC [58], [59] and PPC [60]. Such dynamic stimulus representation may be present even when no elevated activity is detectable during the delay period. In one recent study, the activity of PFC neurons was shown to change dynamically according to the current behavioral rule [61].

### Effective connectivity during working memory

Dynamic changes in functional interactions between neurons, which could allow for such flexible representation of information, have been reported in frontal areas including the dlPFC and the Frontal Eye Field [32], [40], [62]. In recent years, active decorrelation mechanisms have also been suggested, that can alter the strength of functional interaction between neurons [63]. Computational modeling suggests that functional properties of cortical networks, including resistance to interference by distractors during working memory depends on the functional strength of connectivity between neurons, and this can be dynamically modulated by dopamine [21], [26].

Stimulation of dopamine receptors is thought to be vital for regulating the recurrent microcircuitry of the dlPFC [64]–[66], by enhancing excitatory persistent activity to preferred stimulus location of a neuron [22], [67], and inhibiting activity to non-preferred stimulus location to achieve finer spatial tuning during working memory [68]–[70]. Computational studies have predicted that networks involving dopamine inputs are characterized by an enhanced NMDA conductance which facilitates persistent discharges with a higher signal-to-noise ratio [21]–[24]. A balance between excitation and inhibition in a network is crucial for controlling firing pattern during persistent discharge state [27], [30]. Having slower excitation relative to negative feedback can prevent dynamic instability and leads to a better control of recurrent excitation [26], [27]. The slow time constant of NMDA receptors (50–100 ms) is well suited for maintaining the postsynaptic neuron in a depolarized state for a time sufficient for persistent activity to reverberate in the network [28], [29]. Repetitive stimulation saturates the NMDA synaptic current and supports the maintenance of stable persistent activity [27]. Therefore, having a sufficiently high ratio of NMDA/AMPA currents in the local synapses would be a key for this mechanism. Dopamine is one of the factors that can enhance NMDA currents [71]. Some dopaminergic projections and dopamine receptors have also been observed in the PPC, although their relative functional influence has not been investigated as extensively as in the dlPFC [19], [72], [73].

### Functional specializations that support dynamic modulation of connectivity

Anatomical differences between dlPFC and PPC have been indicated that could also account for differential dynamic modulation of synaptic strength. Although dendritic trees of neurons in both areas spread up to more than a millimeter [6], [74], pyramidal neurons in the dlPFC are characterized by the most widespread dendritic trees and largest number of spines of any cortical neurons [75], [76]. This extensive structure could allow dlPFC neurons to connect to a larger number of neurons located more remotely and provide the dlPFC network greater stability. A potential functional consequence of this anatomical difference is that the effective strength of pairwise connections between two neurons in the dlPFC is systematically lower than in the PPC, particularly for neurons located at short distances of each other [31]. Unique interneuron types have also been identified in the dlPFC [77], [78]. Calbindin-containing interneurons have been implicated in a mechanism that increases resistance to noise and distractor interference in a network [79]. Neurons with anatomical [80] and functional [81] properties that fit this model have been observed in greater numbers in the dlPFC than in other cortical areas, including the PPC.

In the present study, we observed differences in intrinsic effective connectivity across the time course of a trial during the behavioral tasks, similar with previous studies [32], [40], [62]. Dynamic changes in effective connectivity were generally uncorrelated with changes in firing rate (Fig. 3). Regardless of whether pairs of neurons shared the same location preference or not, effective connectivity was higher in the PPC than dlPFC during the delay period. Even when both neurons in pairs exhibited significant delay activity, connectivity was lower in the dlPFC compared to the PPC. It seems therefore that, although the potential effects of dopamine or other dynamic factors on facilitating persistent delay activity are greater in dlPFC, their effects may be modest compared to differences in intrinsic connectivity, due to anatomical differences between areas.
